# Epidermal Growth Factor Receptor Mutation Status and Rad51 Determine the Response of Glioblastoma to Multimodality Therapy with Cetuximab, Temozolomide, and Radiation

**DOI:** 10.3389/fonc.2013.00013

**Published:** 2013-02-04

**Authors:** Phyllis Rachelle Wachsberger, Richard Yaacov Lawrence, Yi Liu, Barbara Rice, Constantine Daskalakis, Adam P. Dicker

**Affiliations:** ^1^Molecular Radiation Biology, Department of Radiation Oncology, Thomas Jefferson UniversityPhiladelphia, PA, USA; ^2^Radiation Oncology, Sheba Medical CenterTel Hashomer, Israel; ^3^Pharmacology and Experimental Therapeutics, Thomas Jefferson UniversityPhiladelphia, PA, USA

**Keywords:** GBM, cetuximab, temozolomide, Rad51, radiation therapy, EGFR

## Abstract

**Purpose:** EGFR amplification and mutation (i.e., EGFRvIII) are found in 40% of primary GBM tumors and are believed to contribute to tumor development and therapeutic resistance. This study was designed to investigate how EGFR mutational status modulates response to multimodality treatment with cetuximab, an anti-EGFR inhibitor, the chemotherapeutic agent, temozolomide (TMZ), and radiation therapy (RT).

**Methods and Materials:** *In vitro* and *in vivo* experiments were performed on two isogenic U87 GBM cell lines: one overexpressing wildtype EGFR (U87wtEGFR) and the other overexpressing EGFRvIII (U87EGFRvIII).

**Results:** Xenografts harboring EGFRvIII were more sensitive to TMZ alone and TMZ in combination with RT and/or cetuximab than xenografts expressing wtEGFR. *In vitro* experiments demonstrated that U87EGFRvIII-expressing tumors appear to harbor defective DNA homologous recombination repair in the form of Rad51 processing.

**Conclusion:** The difference in sensitivity between EGFR-expressing and EGFRvIII-expressing tumors to combined modality treatment may help in the future tailoring of GBM therapy to subsets of patients expressing more or less of the EGFR mutant.

## Introduction

Glioblastoma multiforme (GBM) is the most common and aggressive primary adult brain tumor. The standard of care is maximal surgical resection followed by radiation therapy (RT) combined with concurrent and adjuvant temozolomide (TMZ), producing a median survival of only 14.6 months (Stupp et al., 2007).

Molecular targeted agents against key growth factors and signaling pathways in GBM are currently being investigated. In particular, the epidermal growth factor receptor (EGFR) appears to play an important role in tumor growth, survival, and therapeutic resistance (Chakravarti et al., 2002). Amplification of the *EGFR* gene, resulting in overexpression of EGFR protein (Frederick et al., 2000) is seen in 30–50% of GBM cases. Additionally, EGFR proteins are often mutated in GBM. The most common variant, EGFRvIII, has a truncated extracellular domain imparting ligand-independent constitutive activity (Wong et al., 1992).

Erbitux™ (cetuximab) is an anti-EGFR chimeric mouse-human monoclonal antibody approved by The U.S. Food and Drug Administration for colon and head-and-neck cancer. Cetuximab can bind to EGFRvIII as well as EGFR through domain III in the extracellular portion of the receptor, thereby inhibiting downstream signaling pathways (Patel et al., 2007). Unfortunately, clinical trials of cetuximab in GBM have produced overall disappointing results (Neyns et al., 2009). We hypothesized that EGFR mutational status modulates the response to cetuximab, when combined with radiation and temozolomide in the treatment of GBM. *In vitro* and *in vivo* experiments were performed on two isogenic U87 GBM cell lines: over expressing either wildtype (U87wtEGFR) or mutant (U87EGFRvIII) EGFR receptor to test this hypothesis.

## Materials and Methods

### Immunoblot

Western blotting was performed as previously described (Wachsberger et al., 2012). Primary antibodies against EGFR, EGFRvIII, and *O*-6-methylguanine-DNA methyltransferase (MGMT) were obtained from Cell Signaling Technologies (Beverly, MA, USA).

### Animal and tumor model

**Figure 1:**
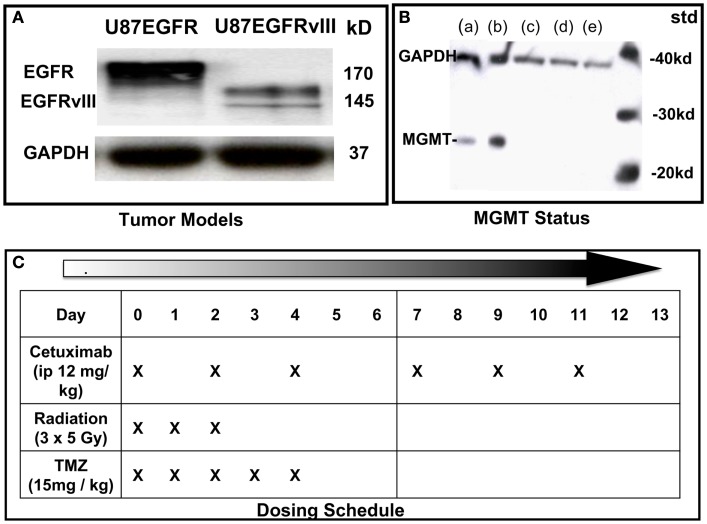
**Characterization of cell lines and dosing schedule**. **(A)** Immunoblot of U87 cells expressing wild type (wt) EGFR or EGFRvIII. **(B)** Immunoblot of MGMT status of U87 transfectants: (a) = LN18 glioma; (b) = MGMT positive control; (c) = U87wtEGFR; (d) = U87EGFRvIII; (e) = U87 parent. **(C)** Dosing schedule. Mice were randomized into eight experimental groups (10–15 animals per group).

U87 GBM cells (American Type Culture Collection), originally lacking expression of EGFR were stably transfected with EGFR or EGFRvIII as previously described (Wang et al., 2006; Figure 1A). Cell suspensions (5 × 10^5^ cells in 100 μL phosphate buffered saline) were implanted subcutaneously (SC) into the right hind limbs of athymic NCR NUM mice (Taconic Farms, Hudson, NY, USA). Tumors were synchronized to be approximately 60 mm^3^ at the start of treatment (day 0) and were measured 3–4 times per week, for up to 6 weeks of follow-up, or until they reached 2,000 mm^3^ (in accordance with IACUC regulations). All animals were randomized among treatment groups. Tumor size was determined by direct measurement with calipers and calculated by the formula: (smallest diameter^2^ × widest diameter)/2.

### Drug and radiation treatment

Cetuximab (supplied by Imclone Pharmaceuticals) was administered i.p. at 12 mg/kg three times a week for 2 weeks, starting on Day 0. TMZ (obtained through Thomas Jefferson University pharmacy) was administered by oral gavage at 15 mg/kg daily on Days 0–4. Irradiation was performed on anesthetized mice using X-rays generated by a PanTak, 310 kVe X-ray machine, 0.25 mm Cu + 1 mm Al added filtration, at 125 cGy per min. Dosimetry was performed by an in-the-beam ionization chamber calibrated against a primary standard. Corrections were made daily for humidity, temperature, and barometric pressure. Mice were anesthetized with a combination of ketamine and acepromazine at a concentration of 37.5 and 0.2 mg/kg, respectively, to provide 25–30 min of sedation. Each mouse was confined in a lead casing with its tumor-bearing leg extended through an opening on the side to allow the tumor to be irradiated locally. Radiation was administered as three daily fractions of 5 Gy each on days 0, 1, and 2. On days when radiation was administered with cetuximab and temozolomide, drugs were given 2 h before radiation. Dosing schedule is shown in Figure 1C.

### Statistical analysis of tumor growth curves

Curves were analyzed via mixed-effects regression, as previously described (Wachsberger et al., 2011). Briefly, this approach fits a random growth curve to each animal’s data and then statistically “averages” these curves within each treatment group to estimate an overall effect for each group. This approach does not depend on an arbitrary endpoint target tumor size, yields generalizable parameters of interest (e.g., average daily tumor growth rate and tumor doubling time), and can investigate treatment interactions. It is also quite powerful since it utilizes the repeated tumor size measurements obtained over the entire study period, while it appropriate handles unbalanced data (i.e., different number of measurements per animal) and the correlation of each animal’s measurements over time. All statistical analyses were conducted in SAS 9.2 (SAS Institute Inc., Cary, NC, USA, 1999–2001).

### Cell viability assay

Cell viability was measured by an MTS assay (Promega, Madison, WI, USA). Exponentially growing cells were plated at 5000 cells/well in a 96-well plate and allowed to incubate for 24 h before treatment with increasing doses of TMZ and the RAD51 inhibitor, B02 (5 μM; Sigma Aldrich, St. Louis, MO, USA) as described in Results. Cells were assayed 24 h following treatment.

### Clonogenic cell survival after radiation, cetuximab, and/or TMZ

Clonogenic cell survival assays was performed with exponentially growing cells in the absence or presence of cetuximab (10 μg/ml) and/or TMZ (10 μM), as follows: cells were plated in T-25 flasks and were irradiated with increasing doses of X-rays with a PanTak 310 keV X-ray machine at 0.25 mm Cu plus 1 mm Al added filtration, at 125 cGy/min. Following irradiation, drugs were removed and flasks were incubated at 37°C for 2 weeks, after which cultures were stained and scored for colony formation. Only colonies of 50 or more cells were counted. Three replicates per dose were studied. Survival curves were generated after normalizing for cell killing by individual drugs alone or in combination. The surviving fraction value was corrected for cellular multiplicity to provide single-cell survival (Sinclair and Morton, 1965). Data were fit to a linear quadratic model for cell survival using GraphPad Prism software (La Jolla, CA, USA). The mean ± SEM from at least three independent experiments were obtained.

### Analysis of Rad51 foci

Cells were fixed 30 min, 4, 6, 24, and 48 h following treatment with 4 Gy X-rays with and without prior incubation (24 h) with TMZ (5 μM). Primary mouse monoclonal anti-Rad51 (Abcam, Cambridge, MA, USA) was added at a dilution of 1:500 in 5% bovine serum albumin (BSA). After incubation and washing, secondary donkey anti-goat (Alexa Fluor 594, Invitrogen, Molecular Probes, Eugene, OR, USA) was added at a 1:500 dilution in 5% BSA-PBS. Rad51 foci were visualized)… on a Zeiss LSM 510 Meta Confocal Microscope (Carl Zeiss Microscope Inc., Thornwood, NY, USA) using a 40X oil immersion lens and analyzed by Image J software provided by NIH. Cells containing nuclei with three or more Rad51 foci were classified as positive for DNA damage. Fifty nuclei were counted for each treatment. Experiments were repeated in triplicate. The Akt inhibitor, BAY 1001931 was provided by Bayer Pharma AG.

## Results

### *In vivo* studies

#### Tolerability and modeling

**Figure 2:**
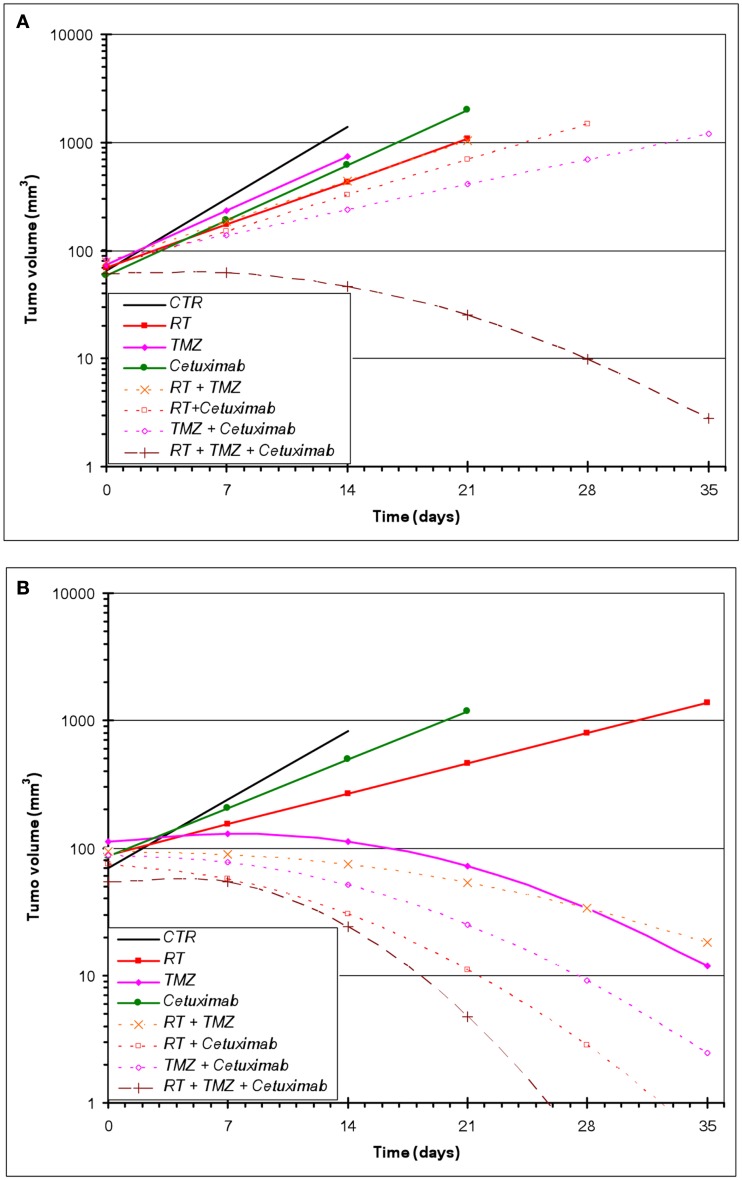
**Estimated geometric mean tumor volume over time after single and combined treatments with RT, cetuximab, or TMZ**. **(A)** U87wtEGFR; **(B)** U87EGFRvIII. Mixed-effects linear regression, as described in Section “Materials and Methods,” was used to model tumor volume as a function of time and treatment. Each treatment group consisted of 10–15 animals.

All treatments were well tolerated in the animals with no observable loss of body weight. For U87wtEGFR-expressing tumor experiments, a linear tumor growth model (on the log-10 scale) fit the data quite well, with the exception of the three-way combination group, for which a quadratic term was necessary (Figure 2A). For the U87EGFRvIII expressers, in five out of the eight groups (TMZ alone, and all combination treatment groups), a linear tumor growth model was not appropriate because of tumor regression in many animals. Therefore, quadratic terms were necessary for these groups (Figure 2B).

#### Effect of radiation, TMZ, and cetuximab on U87wtEGFR tumor xenograft growth

Table 1 summarizes the geometric mean tumor volume (in mm^3^) over time, as well as the tumor growth rate and doubling time, for each treatment group. The control group had an estimated average daily tumor growth rate of 24%, corresponding to an estimated average tumor doubling time of about 3.2 days. Treatment with radiation alone significantly slowed tumor growth compared to the untreated control group (*p* = 0.002), while TMZ alone and cetuximab alone were both marginally better than the controls (*p* = 0.077 and 0.098, respectively).

**Table 1:** **Estimates of the geometric mean tumor volume (in mm^3^) over time, as well as the tumor growth rate and doubling time, by treatment group, for the wtEGFR U87 glioblastoma cell line**.

	Time (days)						%Δ	T_2x_
	0	7	14	21	28	35	
CTR	66	303	1402	*	*	*	24.4	3.2
RT	69	173	433	1084	*	*	14.0	5.3
TMZ	74	235	741	*	*	*	17.9	4.2
Cetuximab	58	190	615	1996	*	*	18.3	4.1
RT + TMZ	79	187	442	1046	*	*	13.1	5.6
RT + cetuximab	71	151	325	697	1494	*	11.5	6.4
TMZ + cetuximab	82	140	239	408	698	1193	8.0	9.0
RT + TMZ + cetuximab	61	63	47	25	10	3	n/a	n/a

*Predicted geometric mean greater than 2,000 mm^3^ (beyond data range).

*%Δ, estimated average rate of increase of tumor volume (% daily change)*.

*n/a, not applicable (tumor shrinkage over time, with estimated average daily tumor size decrease of 1.9% on day 7, 6.3% on day 14, 10.5% on day 21, and 14.5% on day 28)*.

*T_2x_, estimated average doubling time of tumor volume (in days)*.

The two-way combination groups (radiation plus TMZ, radiation plus cetuximab, and TMZ plus cetuximab) had estimated average tumor growth rates between 8 and 13% and were all significantly better than the control group (all *p*-values < 0.001). Radiation combined with TMZ did not show benefit over the corresponding single treatments (*p* = 0.756 against radiation alone and 0.159 against TMZ alone). Radiation plus cetuximab did not show benefit over radiation alone (*p* = 0.383), but was better than cetuximab alone (*p* = 0.038). Finally, TMZ plus cetuximab was significantly better than either radiation alone (*p* = 0.004) or cetuximab alone (*p* = 0.002).

The three-way treatment combination group showed a markedly different tumor growth pattern, with tumors being stable or growing somewhat during the first week and shrinking afterward. The average rate of shrinkage accelerated over time (see Table 1). In 5 out of 12 animals in this group (42%), tumors regressed completely within 4 weeks of treatment initiation. The three-way combination group also seemed better than the cetuximab-only group (*p* = 0.095) and the TMZ plus cetuximab group (*p* = 0.077), which had only one tumor regression each.

#### Effect of RT, TMZ, and cetuximab on U87EGFRvIII tumor xenograft growth

In contrast to U87wtEGFR tumors, linear tumor growth curves fit only the control, radiation-only, and cetuximab-only groups containing EGFRvIII xenografts. The TMZ-only and all combination groups showed a markedly non-linear tumor growth, with tumors being stable initially and then shrinking at varying rates (Figure 2B; Table 2). The control group had an estimated average daily tumor growth rate of 19.3%, corresponding to an estimated average tumor doubling time of about 3.9 days (Table 2). Compared to the untreated controls, radiation alone showed significant slowing of tumor growth (8.2%, *p* = 0.001), but cetuximab alone was only marginally better (13.3%, *p* = 0.074). For the remaining groups, tumor growth rate was not constant over the 4–5 weeks of follow-up (non-linear tumor growth pattern). (Estimated growth rates at days 0, 7, 14, and 21 are shown in Table 2). The combination of radiation, TMZ, and cetuximab had the most pronounced curvature, reflecting an increasingly faster rate of tumor shrinkage toward the end of the follow-up, compared to the other groups. A total of 18 tumors across all groups regressed completely. This number was considerably higher than the number of regressors (five regressors in the three-way combination group) seen in U87wtEGFR tumors. In summary, treatment effects for the EGFRvIII tumors were more dramatic, with tumor shrinkage seen in the three-way combination and all three two-way combination treatment groups as well as the TMZ-only group.

**Table 2:** **Estimates of the geometric mean tumor volume (in mm^3^) over time and tumor growth rate, by treatment group, for the EGFRvIII U87 glioblastoma cell line**.

	Tumor volume						%Δ			
	Time (days)						Time (days)			
	0	7	14	21	28	35	0	7	14	21
CTR	70	241	827	*	*	*		19.3		
RT	88	153	265	460	797	1383		8.2		
TMZ	112	130	112	72	34	12	4.4	0.0	−4.2	−8.2
Cetuximab	86	206	493	1182	*	*		13.3		
RT + TMZ	94	90	75	54	34	18	0.4	−1.6	−3.6	−5.5
RT + cetuximab	75	57	30	11	**	**	−1.2	−6.3	−11.0	−15.6
TMZ + cetuximab	88	78	51	25	**	**	0.4	−3.8	−7.8	−11.6
RT + TMZ + cetuximab	55	54	24	**	**	**	5.9	−5.7	−16.0	−25.1

*Predicted geometric mean greater than 2,000 mm^3^ (beyond data range).

**Predicted geometric mean less than 10 mm^3^ (tumor regression).

*%Δ, estimated average rate of increase or decrease of tumor volume (% daily change)*.

### In vitro studies of U87EGFR and U87EGFRvIII-expressing cells

In order to understand the greater response of the U87EGFRvIII tumors to treatments, especially with TMZ and TMZ in combination therapy, we examined U87wtEGFR and U87EGFRvIII cells with regard to: (1) MGMT status; (2) DNA damage and repair ability; (3) cell viability and (4) clonogenic survival.

#### MGMT status

U87EGFR and U87EGFRvIII transfectants were similar in that they lacked expression of the DNA-repair protein, MGMT, which prevents cross-linking of double-stranded DNA by TMZ (Figure 1B).

#### Rad51 foci assays

**Figure 3:**
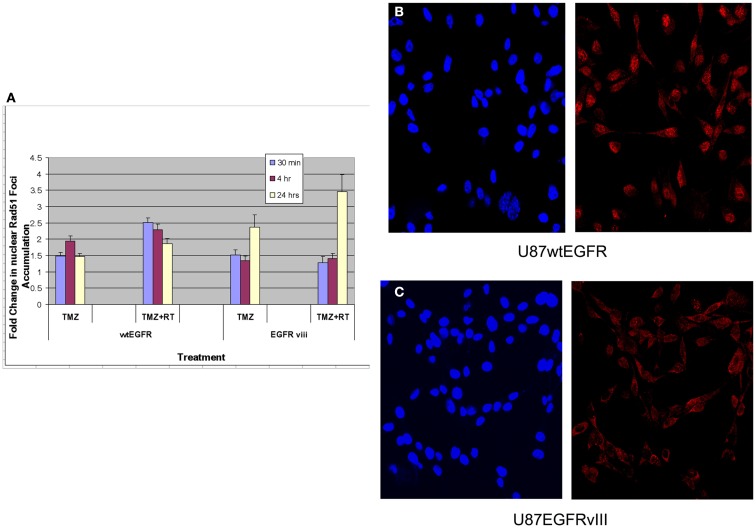
**Effect of TMZ and/or RT on Rad51 foci formation in the nuclei of U87wtEGFR and U87EGFRvIII cells**. Fold-change in nuclear Rad51 foci relative to control levels after treatments with TMZ (20 μM) and/or RT (4 Gy). The mean ± SEM from three independent experiments were obtained with three replicates per experiment. **(A)** Fold-change in nuclear Rad51 foci relative to control levels following TMZ and/or RT treatment. **(B,C)** Micrographs showing DAPI staining of nuclei (left panel) and Rad51 staining (right panel) 4 h following TMZ treatment of U87wtEGFR and U87EGFRvIII respectively. Magnification: 400×.

Previous studies have linked TMZ sensitivity to efficacy of homologous recombination repair (HRR) of DNA damage (Tsaryk et al., 2006). Since Rad51 is a key player in the HRR pathway, studies quantifying Rad51 foci formation and disappearance in the nuclei of EGFR and EGFRvIII cells were made following TMZ and/or RT treatment. Rad51 foci were detectable in nuclei 30 min, 4 and 24 h following treatment with 4 Gy and/or TMZ in both transfectants (Figure 3A). For U87wtEGFR, the number of positive cells exhibiting nuclear Rad51 foci following treatment with TMZ alone was highest 4 h after treatment, exhibiting a 1.9-fold increase over that of control. After 24 h, the number decreased to 1.5-fold. After treatment with TMZ and radiation, the number of nuclear Rad51 foci was highest 30 min after treatment, resulting in a 2.5-fold increase over control. After 24 h, the number decreased to 1.9-fold. (*p* < 0.05 for all comparisons with control).

For U87EGFRvIII, the number of positive cells exhibiting nuclear Rad51 foci following treatment with TMZ was highest at 24 h, exhibiting a 2.4-fold increase over that of control. This change was significantly higher than the 1.5-fold-change observed for EGFR tranfectants following 24 h of treatment (*p* = 0.003). Treatment with TMZ combined with radiation resulted in a maximal increase of Rad51 foci at 24 h [3.5-fold over that of control (Figure 3A)]. Rad51 foci were down to control levels (data not shown) 48 h after treatment.

**Figure 4:**
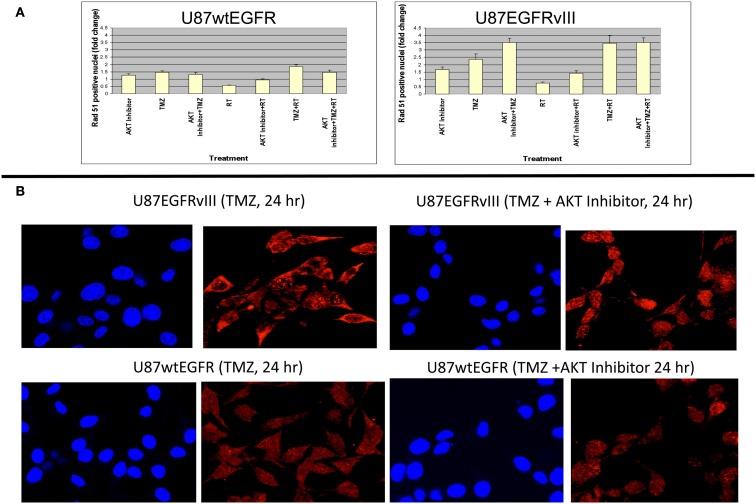
**Effect of Akt inhibitor (BAY 1001931) on nuclear accumulation of Rad51 foci 24 h following TMZ treatment in U87wtEGFR and U87EGFRvIII cells**. **(A)** Fold-change in nuclear Rad51 foci relative to control levels. **(B)** Micrographs showing DAPI staining of nuclei and Rad51 staining 24 h following TMZ treatment (20 μM) with and without Akt inhibitor [BAY1001931 (500 nM) of U87EGFRvIII (a) and U87wtEGFR (b) respectively. Magnification: 400×.

These data indicate a slower rate of nuclear Rad51 foci accumulation and slower rate of disappearance for the EGFRvIII transfectants compared with wtEGFR transfectants. Immunofluorescent staining revealed that Rad51 was more cytoplasmic than nuclear in U87EGFRvIII cells, compared to U87wtEGFRcells following 4 h treatment with TMZ (Figures 3B,C), indicating a slower translocation of Rad51 into nuclei. Since it was previously shown that Akt1 can inhibit HRR by inducing cytoplasmic retention of Rad51 (Plo et al., 2008), additional experiments were performed with a pan Akt inhibitor, BAY1001931 during treatment with TMZ and/or RT to see if Rad51 translocation into the nucleus was affected differently in U87EGFRvIII vs. U87wtEGFR cells. Figure 4A shows a higher fold accumulation of nuclear Rad51 relative to control in the EGFRvIII nuclei compared to wtEGFR nuclei following Akt inhibitor treatment; Figure 4B shows immunofluorescent images of increased Rad51 nuclear staining compared to control following Akt inhibitor treatment in the EGFRvIII transfected cells whereas Rad51 nuclear staining does not change following Akt inhibitor treatment in the wtEGFR-transfected cells. These data suggest that HRR after TMZ treatment in EGFRvIII-transfected cells is retarded because of Akt-activated sequestration of Rad51 in the cytoplasm.

#### Effect of TMZ and TMZ in combination with RAD51 inhibitor (B02) on cell viability in U87EGFR vs. U87EGFRvIII cells

**Figure 5:**
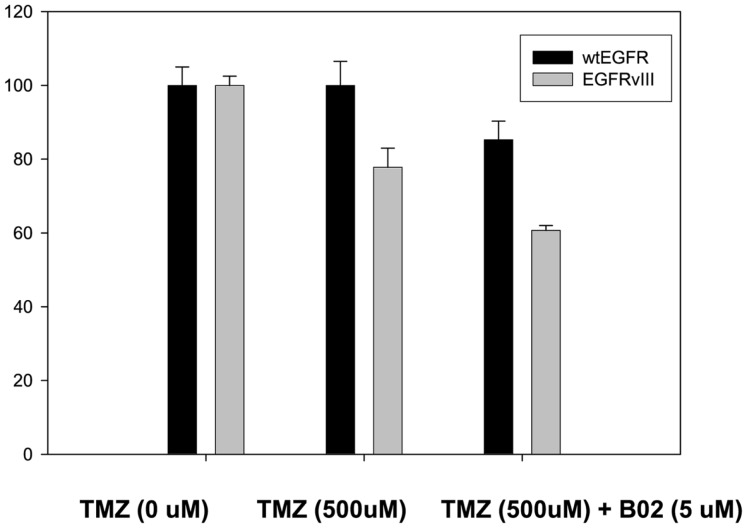
**Effect of TMZ and BO2 on cell viability in U87EGFR vs. U87EGFRvIII cells**. Cell viability was measured by an MTS assay (Promega, Madison, WI, USA) as described in Section “Materials and Methods,” with four replicates per treatment. Experiments were repeated in triplicate.

In order to confirm the increased sensitivity of U87EGFRvIII tumors to TMZ seen *in vivo* (Figures 2A,B), cell viability assays were performed for U87EGFR and U87EGFRvIII cells in the presence of TMZ (500 μM; Figure 5). It can be seen that U87EGFRvIII cells were significantly more sensitive to TMZ (500 μM; *p* < 0.01 for all concentrations). In addition, Rad51 inhibition by B02 increased sensitivity in U87EGFRvIII cells over that of wtEGFR cells (*p* < 0.05).

### Clonogenic cell survival after radiation, TMZ, and/or cetuximab

**Figure 6:**
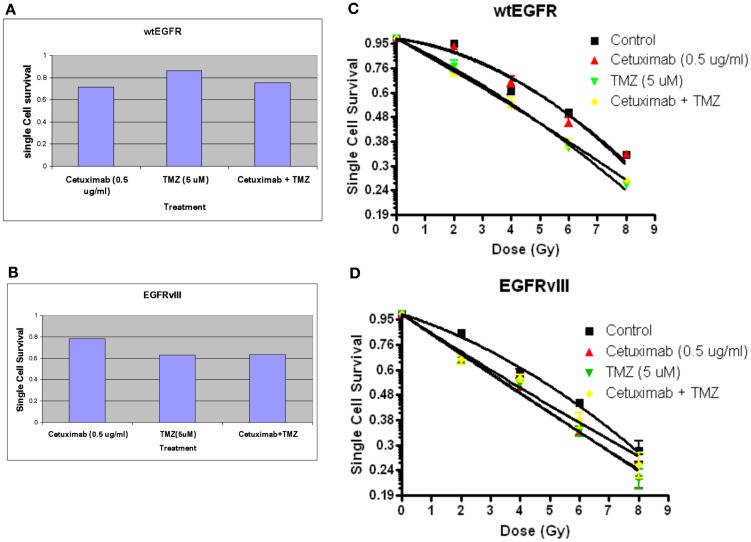
**Effect of TMZ and/or cetuximab on clonogenic cell survival and radiosensitivity in U87EGFR vs. U87EGFRvIII cells**. Cetuximab and/or TMZ were applied to exponentially growing cells 24 h prior to irradiation. **(A,B)** effect of TMZ and/or cetuximab on clonogenic cell survival; **(C,D)** relative radiosensitivities of U87wtEGFR and U87EGFRvIII in the presence of TMZ and/or cetuximab. The mean ± SEM from at least three independent experiments were obtained.

Figures 6A,B indicate the toxicities for cetuximab, TMZ, and cetuximab and TMZ in combination normalized to the plating efficiencies for wtEGFR (P.E. = 0.43) and EGFRvIII (P.E. = 0.36). Figures 6C,D demonstrate that TMZ alone and in combination with cetuximab significantly radiosensitized both transfectants (*p* < 0.001 for TMZ/cetuximab vs. control). There was no significant difference in radiosensitization by TMZ between the transfectants under the conditions of this assay. Cetuximab enhanced radiosensitization in EGFRvIII transfectants (*p* < 0.002 vs. control) but not in wtEGFR.

## Discussion

This study demonstrated that U87EGFRvIII-expressing tumors are more sensitive than U87wtEGFR-expressing tumors to treatment with TMZ alone and TMZ in combination with cetuximab and/or RT. Factors that may contribute to TMZ sensitivity include: a high proliferation rate (Roos et al., 2009); methylation status of the repair enzyme, MGMT (Esteller et al., 2000); efficacy of DNA repair (Kil et al., 2008). With regard to proliferation rate, the doubling time of U87EGFRvIII tumors was actually slower than that of U87wtEGFR tumors in this study, therefore, the increased sensitivity to TMZ observed in these tumors compared to U87wtEGFR tumors could not be explained by proliferation rate differences. With regard to methylation status, U87 GBM cells are known to have hypermethylated MGMT promoters (Hermes et al., 2008) and therefore lack detectable MGMT expression on western blots. Likewise, both U87wtEGFR and U87EGFRvIII tumors derived from the parent U87 cells lacked detectable MGMT expression (data not shown). Consequently, MGMT status could not explain the high sensitivity to TMZ that was seen in U87EGFRvIII tumors.

With regard to efficacy of DNA repair, it has been reported that TMZ can inhibit DNA DSB repair. However, it is not known if one or both of the two key complementary DNA DSB repair pathways, non-homologous end-joining (NHEJ) or HRR are involved in this inhibition. EGFRvIII-expressing cells were shown to accelerate repair of radiation-induced DNA DSBs through upregulation of DNA-PKc, the catalytic subunit of DNA-PK, a key enzyme in the NHEJ pathway (Mukherjee et al., 2009). Although, NHEJ is a very active repair pathway in EGFRvIII following radiation, it is not apparently related to TMZ sensitivity (Roos et al., 2009). There is evidence, however, that HRR may play a role in TMZ sensitivity (Tsaryk et al., 2006). A deficiency in HRR signaling via XRCC2 or other repair enzymes involved in HRR (i.e., Rad51, Rad52, Brca2) might explain increased sensitization to TMZ in EGFRvIII-expressing tumors, especially in light of studies with XRCC2- and Brca2-deficient cells (Tsaryk et al., 2006; Roos et al., 2009) showing increased sensitization to TMZ. We examined the formation of Rad51 foci to see if HRR is defective in EGFRvIII-expressing cells. We found a slower rate of accumulation and slower rate of disappearance of nuclear Rad51 foci in U87EGFRvIII vs. U87wt/EGFR transfectants. In addition, Rad51 staining was largely perinuclear or cytoplasmic in U87EGFRvIII cells compared to U87wtEGFR cells 4 h following TMZ treatment. Previous studies indicated that EGFRvIII-expressing cells constitutively activate phosphatidylinositol 3-Kinase (PI3-K) and Akt1 (Moscatello et al., 1998), and that Akt1 can inhibit HRR by inducing cytoplasmic retention of Rad51 (Plo et al., 2008). Our current study found that inhibition of Akt can increase the rate of nuclear foci accumulation of Rad51 in the EGFRvIII cells compared to EGFR cells and that Rad51 inhibition can heighten sensitivity of EGFRvIII to TMZ. It is therefore suggested that defective HRR may contribute to the heightened sensitivity of EGFRvIII tumors to TMZ observed in this study.

This study also demonstrated that U87EGFRvIII tumor growth was very sensitive to dual treatment with cetuximab plus radiation, causing tumor regressions. Previous investigators determined that gefitinib, a receptor tyrosine kinase inhibitor of EGFR can reduce radioresistance in EGFRvIII-expressing mouse astrocytes through attenuation of DNA dsb repair (Mukherjee et al., 2009). The present study does indicate increased radiosensitization in the presence of cetuximab in U87EGFRvIII compared to U87EGFR *in vivo* and *in vitro*. It is concluded that blocking EGFR/EGFRvIII by cetuximab alone will most likely not translate into a clinical benefit, whereas incorporation of EGFR inhibitor signaling into a combined modality approach will more likely lead to improvement of GBM tumor control. Additionally, the difference in sensitivity between EGFR-expressing and EGFRvIII-expressing tumors to triple modality treatment observed in this study appears to be largely the result of increased TMZ sensitization as well as increased sensitivity to cetuximab and radiation in EGFRvIII tumors and may contribute to individual variation in response to treatment, depending on the level of EGFRvIII expression. Of direct relevance to the present study are the recent clinical findings that patients whose GBM tumors express EGFRvIII have prolonged survival after radiation and chemotherapy with TMZ (Montano et al., 2011). The same study demonstrated *in vitro* that GBM cells lacking EGFRvIII are more resistant to TMZ. Our current study may help in future tailoring of GBM therapy to subsets of patients expressing more or less of the EGFR mutant.

## Conflict of Interest Statement

The authors declare that the research was conducted in the absence of any commercial or financial relationships that could be construed as a potential conflict of interest.
